# Complete Destruction of Both Membrana Tympani; Application of Artificial Drum with Great Benefit

**Published:** 1869-11

**Authors:** 


### Complete Destruction of both Membrana Tympani; Application of Artificial Drum with Great Benefit.

J. Orne Green, M.D., Physician to the Department for Diseases of the Ear, Boston City Hospital, reports the following case: A fine, healthy man, æt. 29, had scarlet fever, when four years old, and has had ever since a continuous, offensive otorrhœa on each side. On examination, the right membrana tympani is entirely destroyed; the hammer remains, very much drawn in, and just below it the mucous membrane of the tympanum is red, irregularly swollen, and in one spot a white mass of calcareous deposit is seen and felt on it; right Eustachian tube pervious. The left ear exhibits almost the same appearances, except that the membrane of the tympanum is perfectly smooth and of a light pink color; no remnants of the membrane tympani to be seen; by Valsalva's experiments there is a distinct perforation-whistle. The watch is heard one second in each ear, the voice about five minutes. Under the use of an iodine instillation, the swelling was so much reduced in the right ear that he could blow air easily through it; the otorrhœa was diminished by the astringent, and finally checked by the use of talc.

Artificial drums were then applied to each ear in the same way as in the preceding case, and to his great gratification his hearing was greatly improved, so that he could easily carry on a conversation with a person twenty feet distant. He was taught to introduce them himself, and before he left for his home in the West could adjust them easily, and with great benefit to the hearing.—*Boston Medical and Surgical Journal.*

